# Supplementary figures and images for: Aβ oligomers trigger necroptosis-mediated neurodegeneration via microglia activation in Alzheimer’s disease
Source: Acta Neuropathol Commun. 2022 Mar 9;10:31. doi: 10.1186/s40478-022-01332-9 (PMC8908658; doi:10.1186/s40478-022-01332-9)

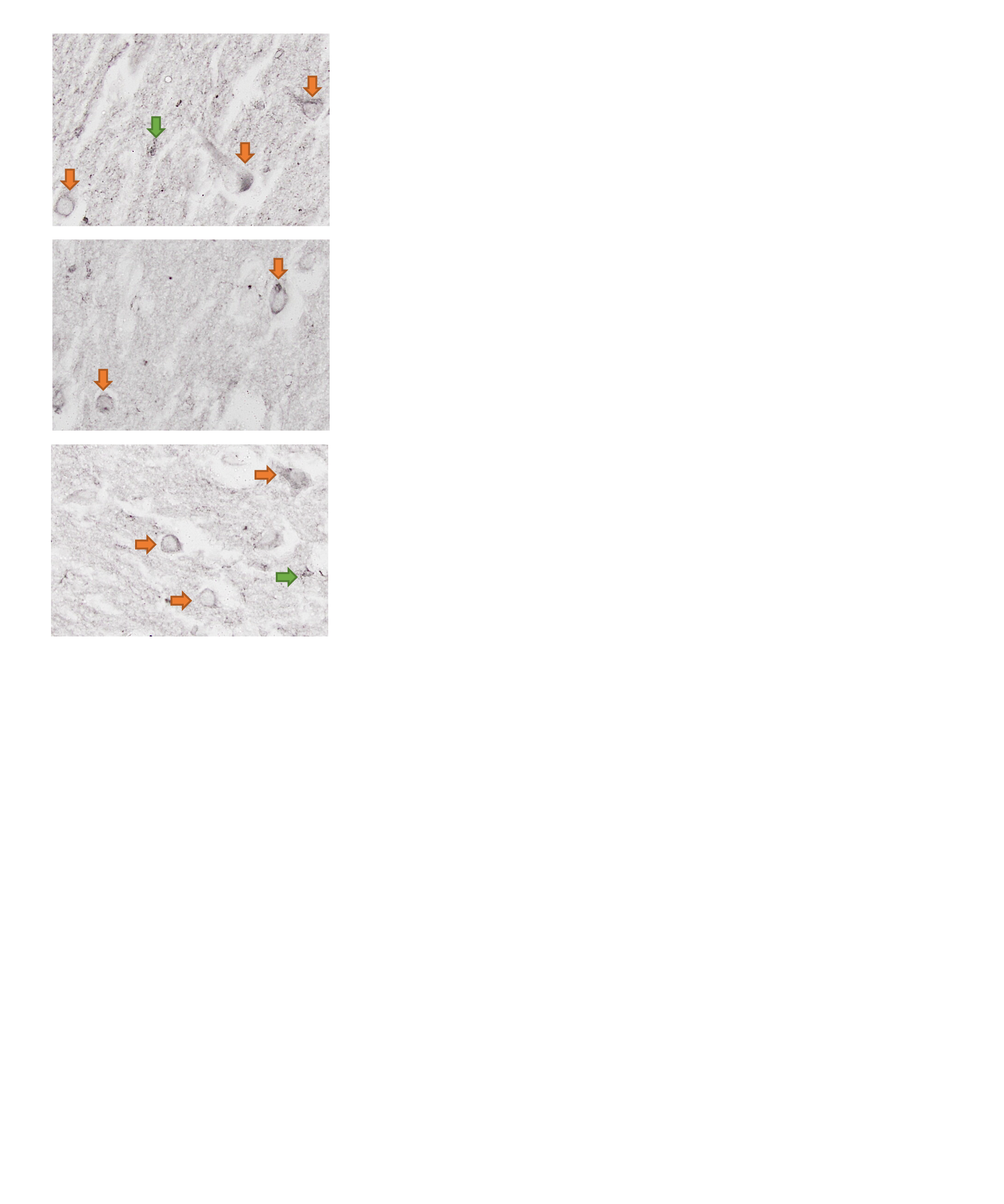

Supplement: Supplementary file 1 — Additional file 1: Fig. S1. Validation of the pMLKL immunoreactivity by DAB staining. Representative micrographs of hippocampal brain areas of AD patients (n = 3) immunostained with anti-pMLKL antibody. Orange arrows indicate pMLKL-positive neurons, and green arrows show pMLKL-positive microglia. Magnification, 20X. [file 40478_2022_1332_MOESM1_ESM.tif]

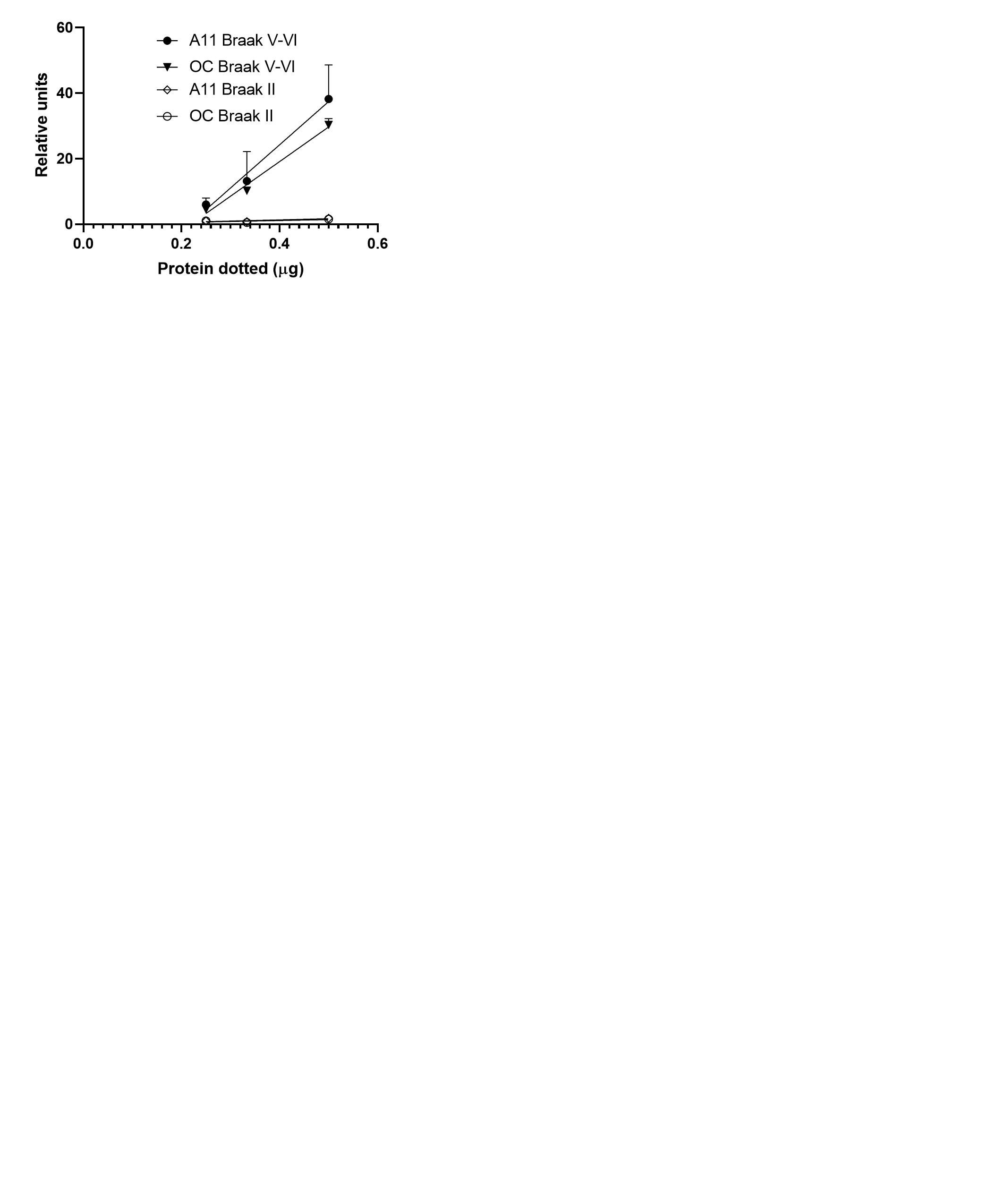

Supplement: Supplementary file 2 — Additional file 2: Fig. S2. Coefficient of variation of the dot-blot assays. The coefficient of variation (CV) was calculated using triplicates of Braak V-VI samples. A CV of 11.63% and 15.06% was determined for OC and A11, respectively. [file 40478_2022_1332_MOESM2_ESM.tif]

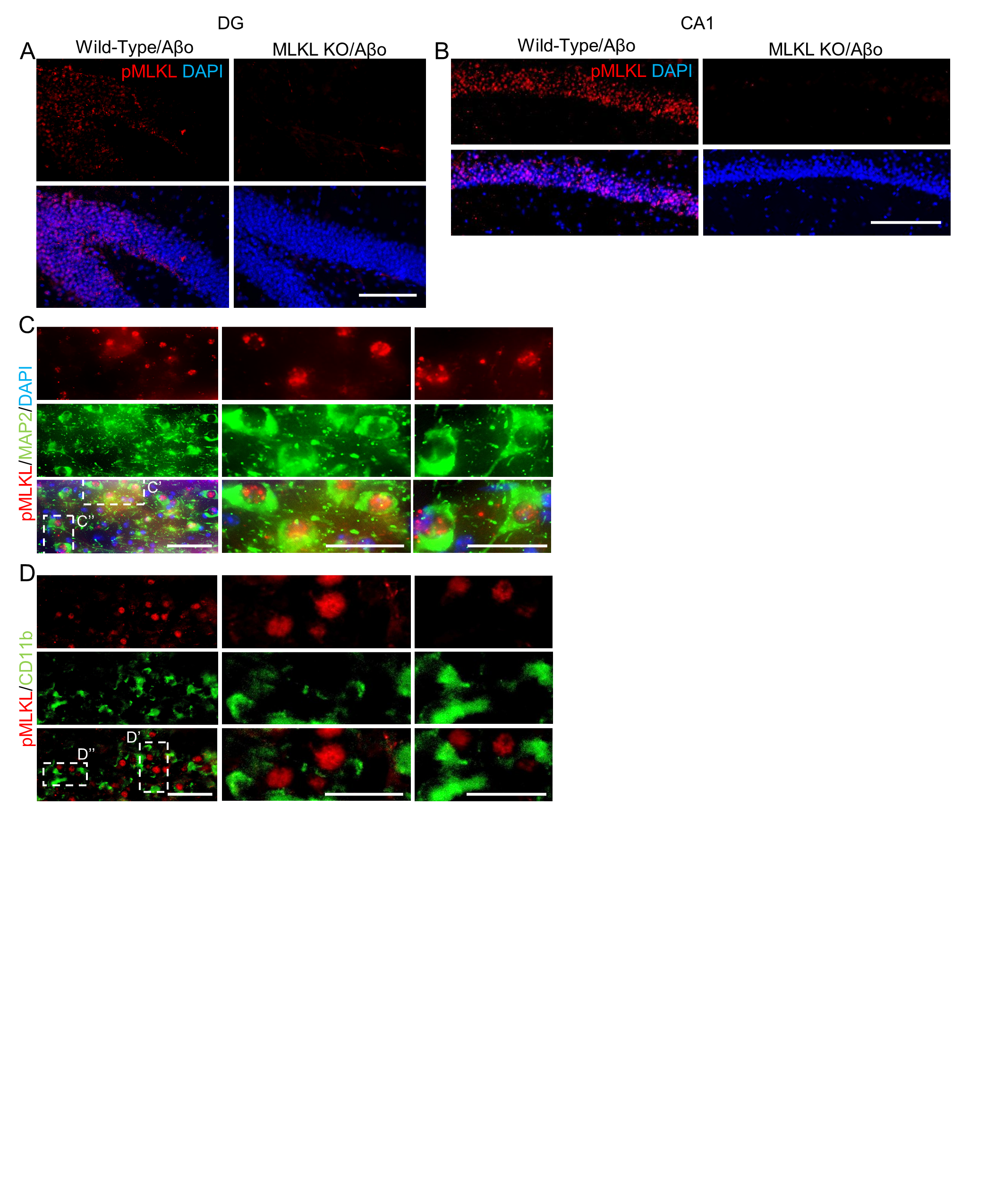

Supplement: Supplementary file 3 — Additional file 3: Fig. S3. Validation of the pMLKL antibody and assessment of cell specificity. Representative micrographs of (A) the dentate gyrus (DG) and (B) CA1 brain regions from wild-type and MLKL knockout mice subjected to intracerebral injection of Aβo, immunostained with anti-pMLKL antibody (scale bar, DG: 100 μm, CA1: 150 μm). (C, D) Representative images of the hilus of wild-type mice treated with Aβo, labeled with the indicated antibodies (scale bar, 50 μm and 25 μm for magnifications). [file 40478_2022_1332_MOESM3_ESM.tif]

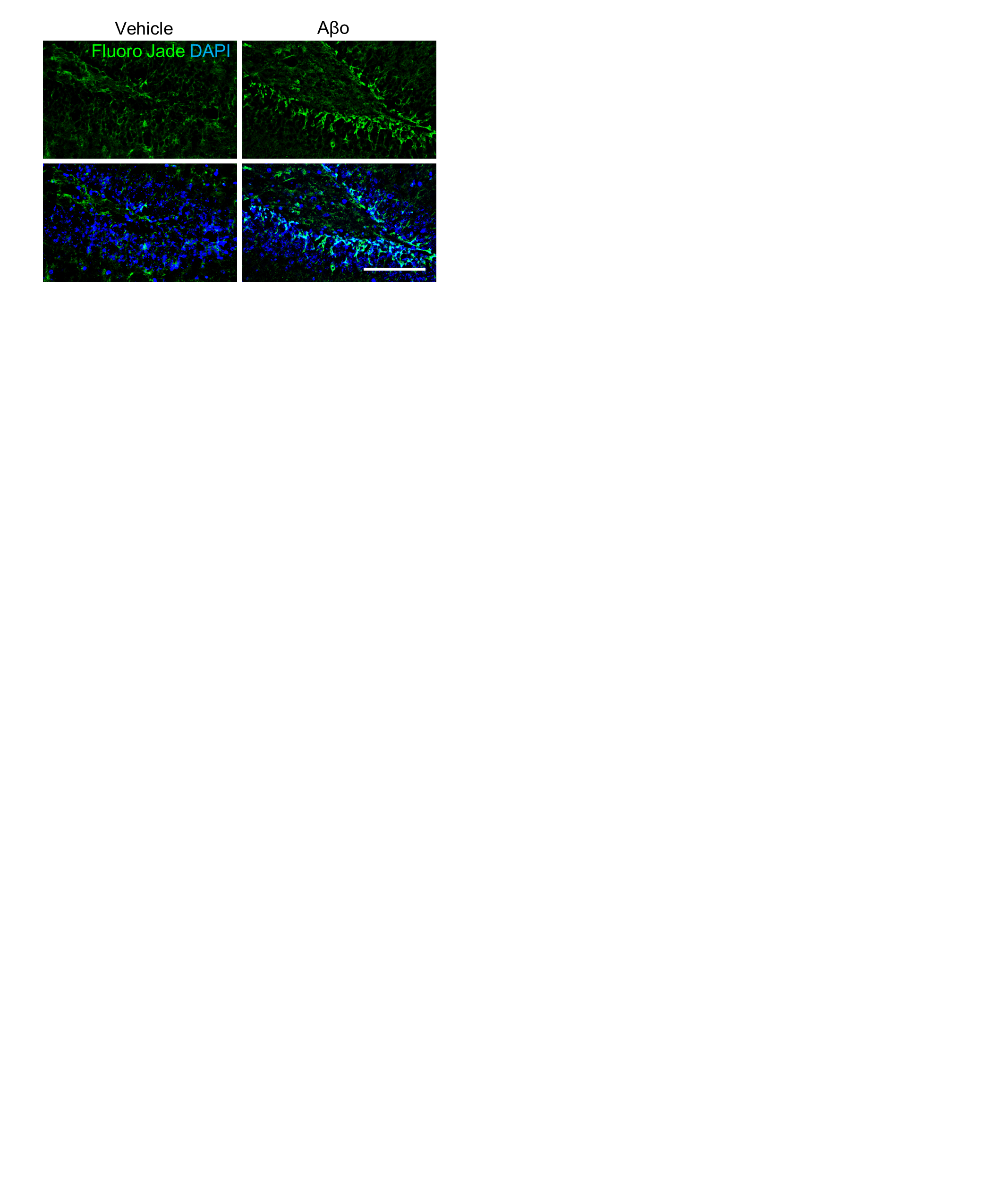

Supplement: Supplementary file 4 — Additional file 4: Fig. S4. Intracerebral administration of Aβo elicits neurodegeneration in wild-type mice. Representative micrographs of brain sections from wild-type mice treated as indicated in the images, stained with Fluoro-Jade C (scale bar, 150 μm). [file 40478_2022_1332_MOESM4_ESM.tif]

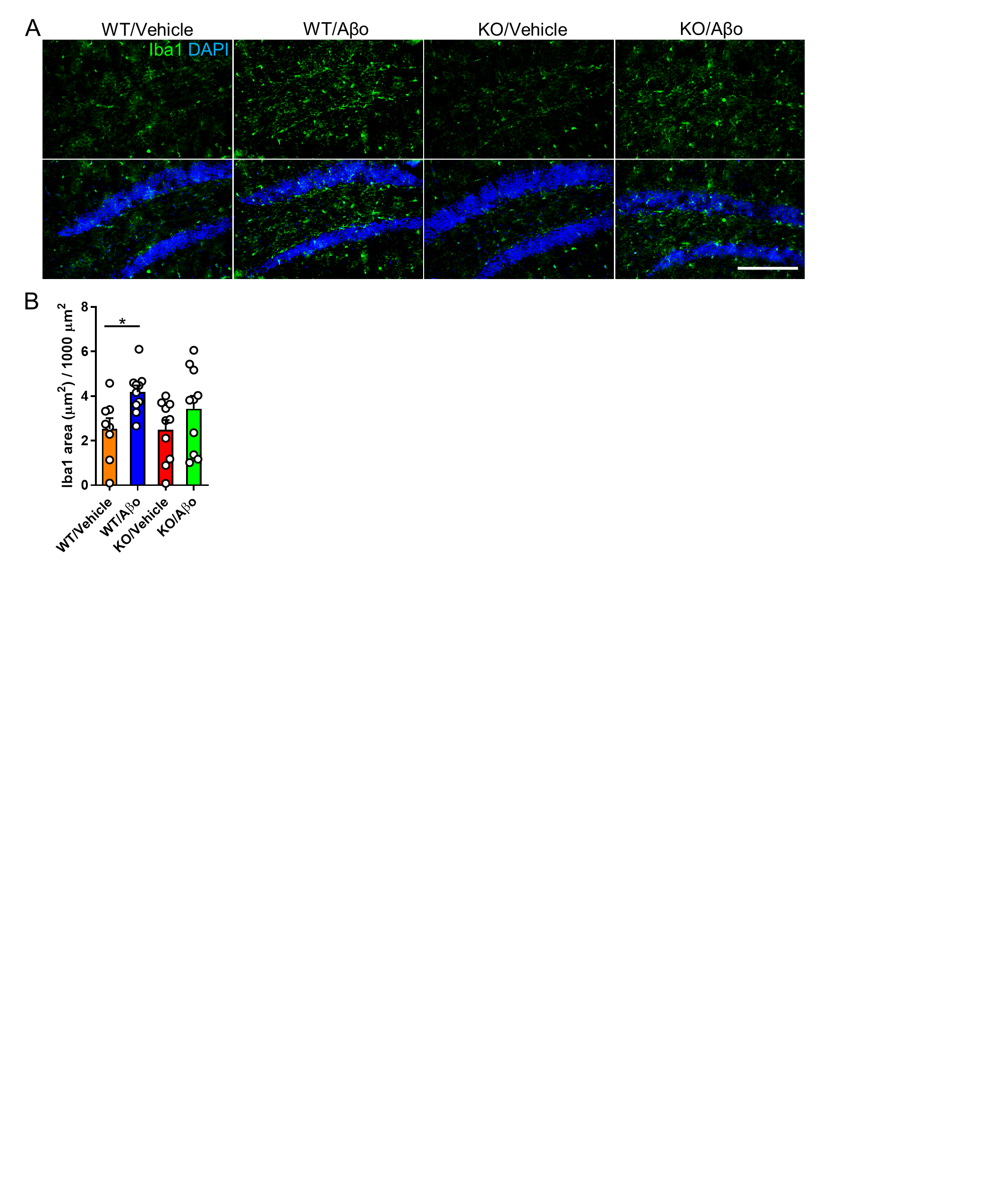

Supplement: Supplementary file 5 — Additional file 5: Fig. S5. MLKL inhibition attenuates Aβo-induced microgliosis in mice. (A, B) Representative micrographs and quantitative analysis of brain sections from wild-type and Mlkl knockout mice treated as indicated in the images, immunostained with anti-Iba1 antibody (scale bar, 200 μm). The data are presented as mean ± S.E.M. and were analyzed by one-way ANOVA followed by Bonferroni post-test. *p < 0.05. [file 40478_2022_1332_MOESM5_ESM.tif]

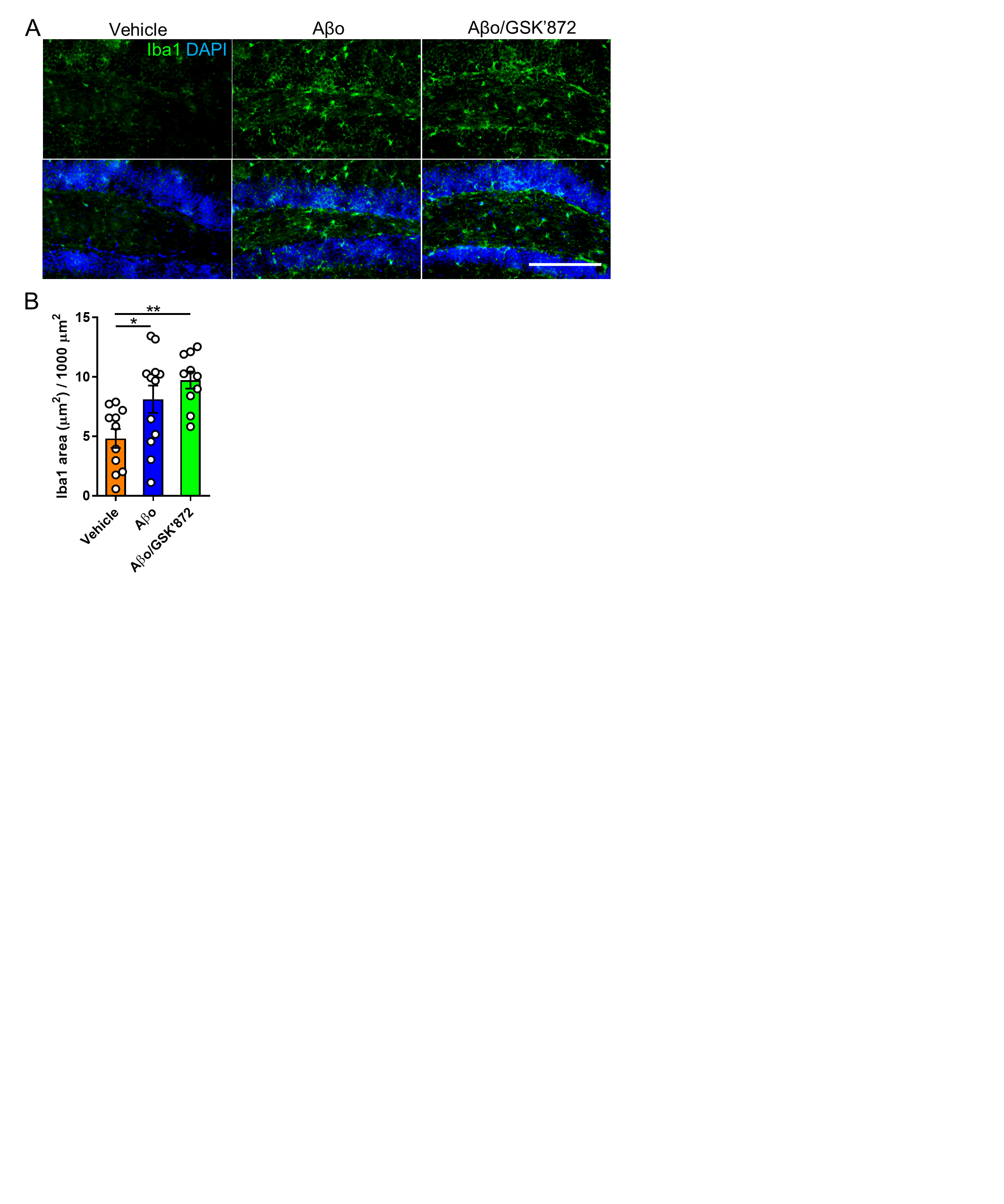

Supplement: Supplementary file 6 — Additional file 6: Fig. S6. RIPK3 inhibition does not alter Aβo-induced microgliosis in wild-type mice. (A, B) Representative micrographs and quantitative analysis of brain sections from wild-type mice treated as indicated in the images, immunostained with anti-Iba1 antibody (scale bar, 200 μm). The data are presented as mean ± S.E.M. and were analyzed by one-way ANOVA followed by Bonferroni post-test. *p < 0.05; **p < 0.01. [file 40478_2022_1332_MOESM6_ESM.tif]

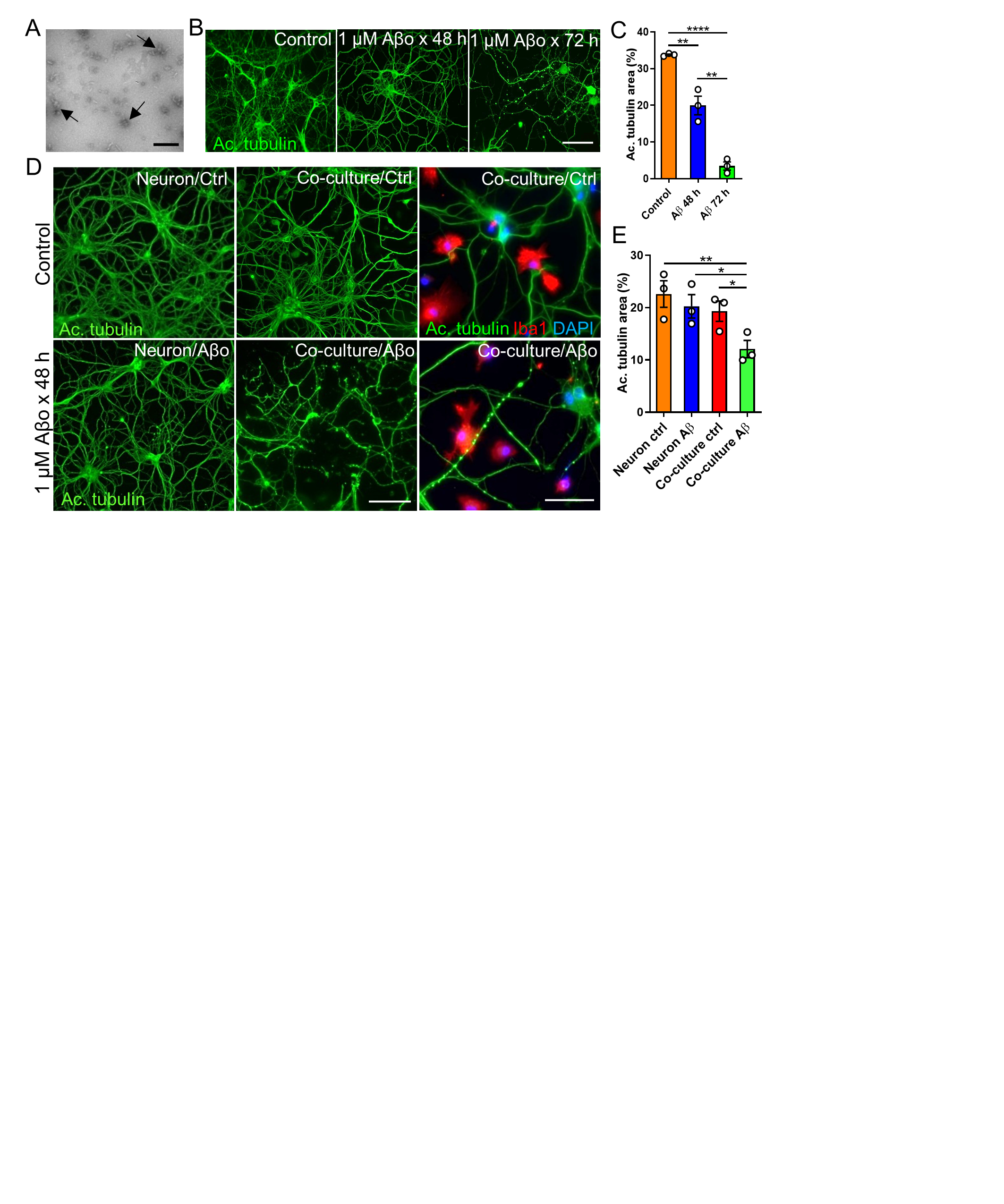

Supplement: Supplementary file 7 — Additional file 7: Fig. S7. Aβo neurotoxicity is worsened when microglial cells are present. (A) To characterize Aβo, a negative stain with 1% uranyl acetate was performed followed by electron microscopy analysis. Arrows indicate small oligomeric structures (scale bar, 200 nm). (B, C) Representative images and quantitative analysis of neurons treated as indicated, immunostained with anti-acetylated tubulin antibody (scale bar, 100 μm). (D, E) Representative micrographs and quantitative measurement of neurons and neuron/microglia cocultures treated as indicated, immunolabeled with anti-acetylated tubulin and anti-Iba1 antibodies as specified in the images (scale bar, 100 μm in the middle panel; 50 μm in the right panel). Each experiment was performed at least three independent times, with three replicates per condition each time. The data are presented as mean ± S.E.M. and were analyzed by one-way ANOVA followed by Bonferroni post-test. *p < 0.05; **p < 0.01; ****p < 0.0001. [file 40478_2022_1332_MOESM7_ESM.tif]

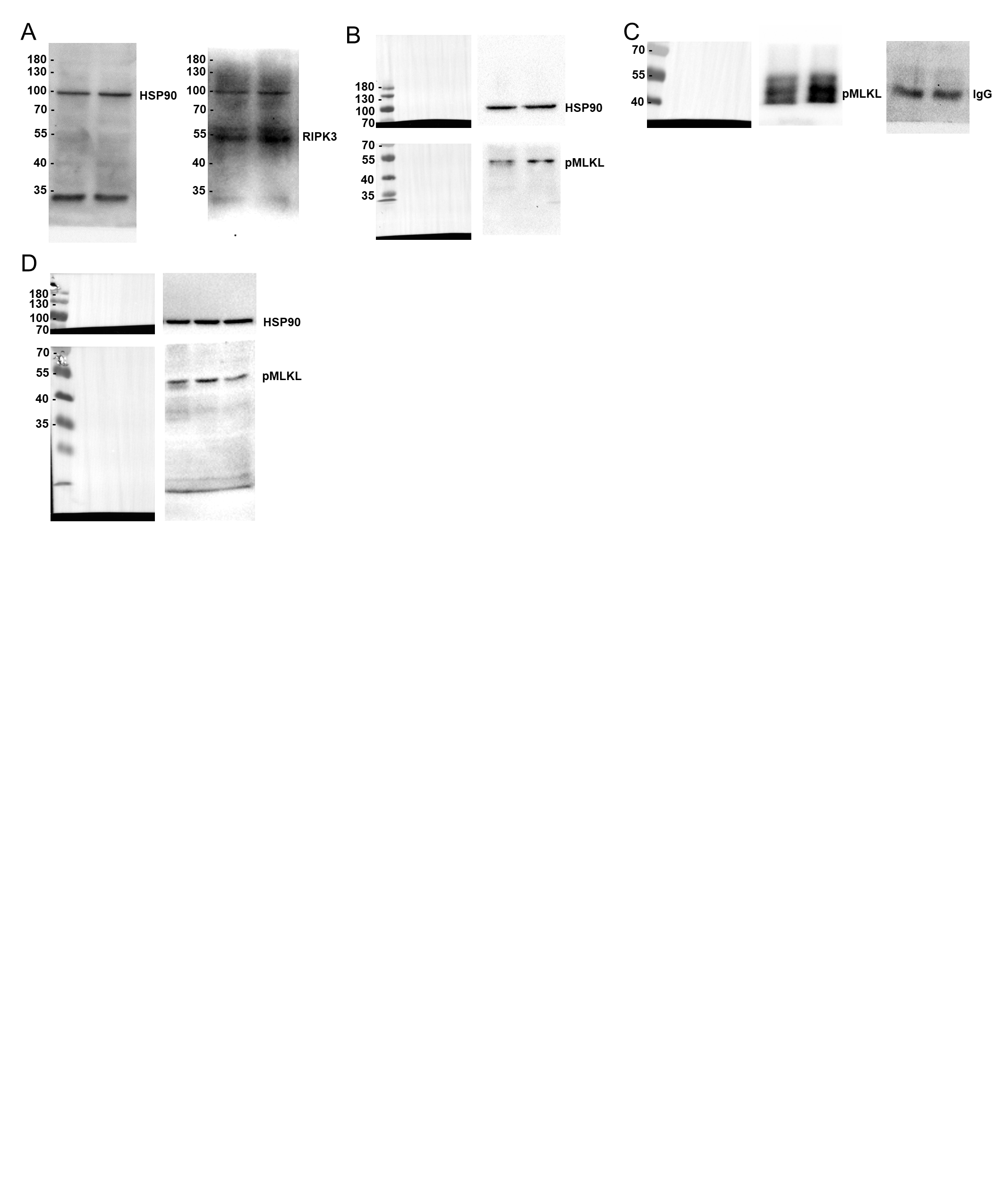

Supplement: Supplementary file 8 — Additional file 8: Fig. S8. Full blots of pMLKL and RIPK3 western blots. (A) The membrane was first probed with an anti-hsp90 antibody and then incubated with anti-RIPK3. (B) The membrane was cut, and each piece was incubated with the indicated antibody. Left panel: colorimetric image showing protein ladder. (C) The membrane was cut and incubated with an anti-pMLKL antibody. Following stripping, and after confirming that no bands could be visualized with ECL, membranes were blocked, and detection of the IgG was done. Left panel: colorimetric image showing protein ladder. (D) The membrane was cut, and each piece was incubated with the indicated antibody. Left panel: colorimetric image showing protein ladder. [file 40478_2022_1332_MOESM8_ESM.tif]

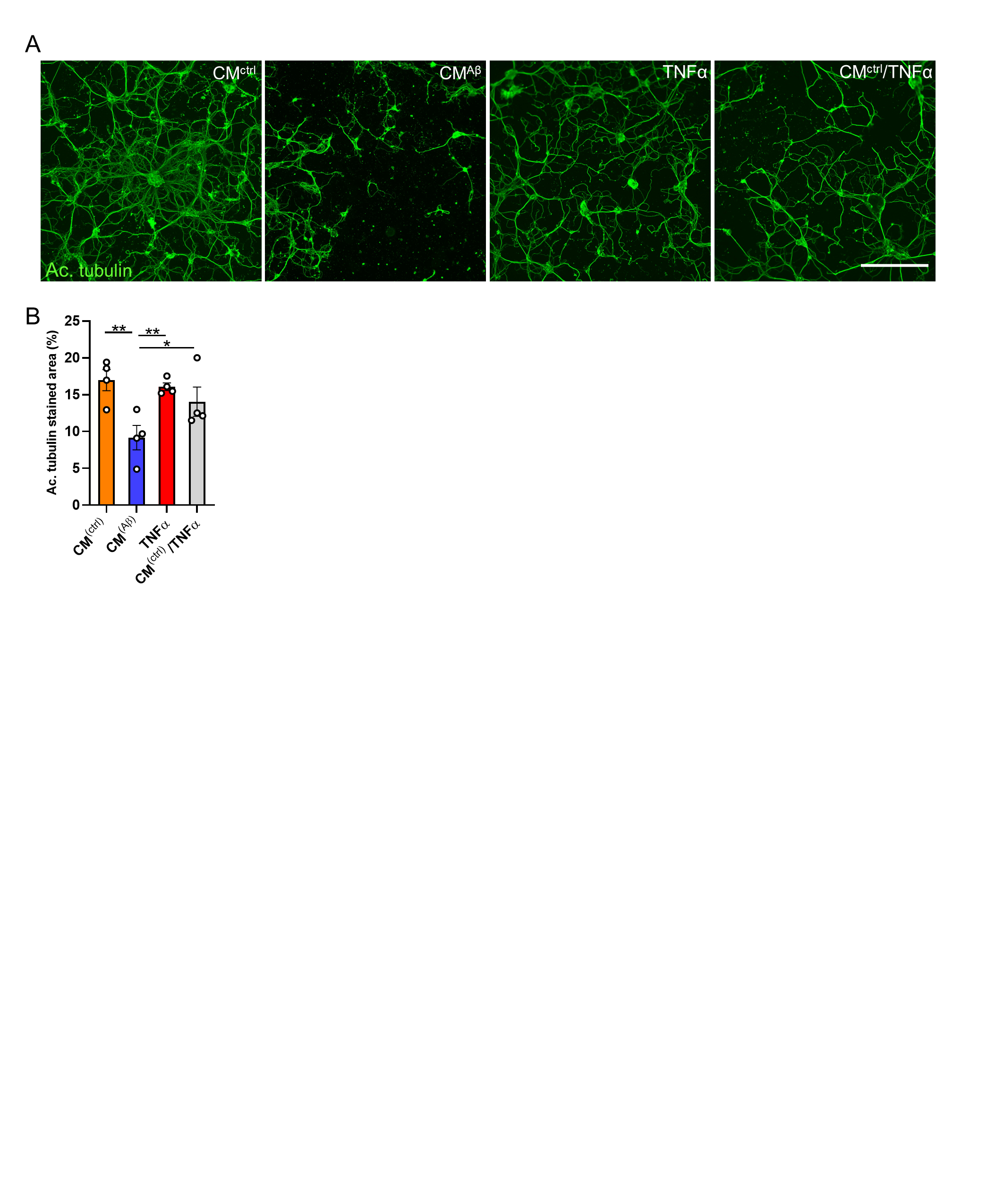

Supplement: Supplementary file 9 — Additional file 9: Fig. S9. TNF-α alone is not sufficient to trigger neuronal necroptosis. (G, H) Representative micrographs and quantitative measurements of neurons treated for 72 h with CMctrl, CMAβ, TNF-α, and with CMctrl/TNF-α, immunolabeled with anti-acetylated tubulin antibody (scale bar, 200 μm). The data are presented as mean ± S.E.M. and were analyzed by one-way ANOVA followed by Bonferroni post-test. *p < 0.05; **p < 0.01. [file 40478_2022_1332_MOESM9_ESM.tif]
